# 3D Electrochemical Sensor and Microstructuration Using Aerosol Jet Printing

**DOI:** 10.3390/s21237820

**Published:** 2021-11-24

**Authors:** Tiziano Fapanni, Emilio Sardini, Mauro Serpelloni, Sarah Tonello

**Affiliations:** 1 Department of Information Engineering, University of Brescia, 25123 Brescia, Italy; emilio.sardini@unibs.it (E.S.); mauro.serpelloni@unibs.it (M.S.); 2 Department of Information Engineering, University of Padova, 35131 Padova, Italy; sarah.tonello@unipd.it

**Keywords:** voltametric sensors, aerosol jet printing, printed electronics, 3D microstructuration, electrochemical sensors

## Abstract

Electrochemical sensors are attracting great interest for their different applications. To improve their performances, basic research focuses on two main issues: improve their metrological characteristics (e.g., repeatability, reusability and sensitivity) and investigate innovative fabrication processes. In this work, we demonstrate an innovative microstructuration technique aimed at increasing electrochemical sensor sensitivity to improve electrode active area by an innovative fabrication technique. The process is empowered by aerosol jet printing (AJP), an additive-manufacturing and non-contact printing technique that allows depositing functional inks in precise patterns such as parallel lines and grids. The 3D printed microstructures increased the active surface area by up to 130% without changing the substrate occupancy. Further, electrochemical detection of ferro/ferri-cyanide was used to evaluate the sensitivity of the electrodes. This evaluation points out a sensitivity increase of 2.3-fold on average between bare and fully microstructured devices. The increase of surface area and sensitivity are well linearly correlated as expected, verifying the fitness of our production process. The proposed microstructuration is a viable solution for many applications that requires high sensitivity, and the proposed technique, since it does not require masks or complex procedures, turns out to be flexible and applicable to infinite construction geometries.

## 1. Introduction

Electrochemical sensors are attracting increasing interest in the scientific community due to their selectivity, sensitivity, ease of use and low cost [1]. In the literature, there are several application fields that use this kind of sensor, for example, to detect physiological conditions or diseases in humans (including for example drug abuse or dosage, fatigue, diagnoses of hypo- and hyperglycemia, cystic fibrosis) [2], pollutants (e.g., heavy metals, pesticides, antibiotics) both in soil and water [3,4] or to analyze biochemical systems (cell cultures, food samples, biomarkers) [5]. In order to obtain continuous monitoring of the analytes, great attention is required in increasing sensor’s repeatability, reusability and sensitivity as well as long-term stability [6]. 

Sensitivity is an important parameter in each application that requires measuring small variations of the analytes of interest. Specific functionalization techniques are usually employed to enhance sensitivity and selectivity [7]. Selectivity is achieved by means of different materials able to bind analytes and/or catalyze the reactions. The former is usually employed in bio-affinity sensors, where specific receptors such as antibodies, proteins or DNA are immobilized on the electrode surface and selectively bind with the analytes [8]. The catalysis process, otherwise, is often obtained with enzymes that are immobilized on the transducer surface and that are able to ease a reaction from an analyte of interest to an electroactive molecule [9]. A widely accepted method reported in the literature to improve the sensitivity is using nanomaterials due to their high conductivity, high surface area and enzyme-mimetic effects [1,10]. Nanostructures are usually deposed or grown on top of the working electrode (WE) of the sensors [11,12]. 

In addition to bio- and nanofunctionalization, one of the most relevant aspects of the surface of the electrodes that can influence both sensitivity and selectivity of the analysis is the microstructuration [13]. Thus, the micrometric 3D structure of the electrode surface can have a strong impact on analyte–electrode interaction, in terms of sample microfluidic and diffusion and in terms of total surface area available for the reaction [14]. WE surface microstructuration usually aims to produce array patterns or increase the electrode porosity [15]. The latter is usually obtained using electrochemical processes [16] and aims to adsorb analytes, while the former is often based on well-defined geometries such as pillars. Pillar-based structures are well explored in the literature where different parameters such as pillars width, distance, layout and shape are taken into consideration [17,18,19]. Those affect two main parameters: the before-mentioned surface area and the diffusion layer of the analytes. The latter is because 3D features have a non-planar diffusion layer and that can interact with the produced current if the diffusion layers of different 3D features overlap or not [18]. The reported fabrication methods involve different techniques such as direct growth, photolithography, reactive ion etching, template synthesis and 3D printing [20]. The last one is attracting great interest—despite its limited resolution—because it works in environmental conditions, does not require harmful chemicals and has fast prototyping [21].

Among the printing techniques, aerosol jet printing (AJP) was selected for this application due to its advantages over other ones, such as the ample set of ink viscosities, line resolution down to 10 µm, and maskless and non-contact production process on non-planar substrates and 3D objects [22]. Briefly, AJP uses a pneumatic atomizer to produce an ink mist in a carrier gas (atomizer flow). In the virtual impactor, the mist is reduced, removing smaller droplets by means of the exhaust flow. Lastly, the remaining flow (aerosol flow) is focused and accelerated in the nozzle by the sheath flow [23]. In this paper, we present how we implemented and characterized a 3D microstructuration process using this technique to demonstrate a possible method to increase the overall sensitivity of an electrochemical sensor. 

## 2. Materials and Methods

### 2.1. Electrode Design and Fabrication

An electrochemical sensor is composed of three electrodes known as working (WE, carries out the electrochemical event of interest), reference (RE, provides a stable equilibrium potential) and counter (CE, needed to complete the electrical circuit). These sensors relate the flow of electrons to the concentration of electroactive species of interest (analytes) that undergo oxidation or reduction reactions. Electrochemistry uses electrical potentials to change the energy level and thus ease redox reactions. In the literature, several techniques are reported to study these processes, and they are provided simplified, yet accurate, relations to describe the current resulting from the experiments [24,25]. Several examples include Randles−Sevcik (1) and Cottrell (2) equations. The former describes the peak current obtained in cyclic voltammetry (CV), an electrochemical experiment where the potential is scanned at a certain velocity (scan rate, ν). The latter describes the current resulting from a chronoamperometry, an electrochemical experiment where the potential is stepped to a value and then kept fixed for a certain time (t).

(1) $$i_{p}=0.446{\mathrm{nAC}}^{*}{\left(\frac{\mathrm{nF}\nu D}{\mathrm{RT}}\right)}^{\frac{1}{2}}$$

(2) $$i\left(t\right)=\frac{{\mathrm{nFAC}}^{*}\sqrt{D}}{\sqrt{\pi t}}$$

Both these equations depend on constants (Faraday constant F, gas constant R), on reaction dependent parameters (number of electrons that are involved n, analyte diffusion coefficient D, absolute temperature T, analyte concentration in the bulk solution C^*^) and from the WE area (A). This last parameter is the only one that we can control during the design process, and conveniently, it is directly proportional to the output current.

**Figure 1:**
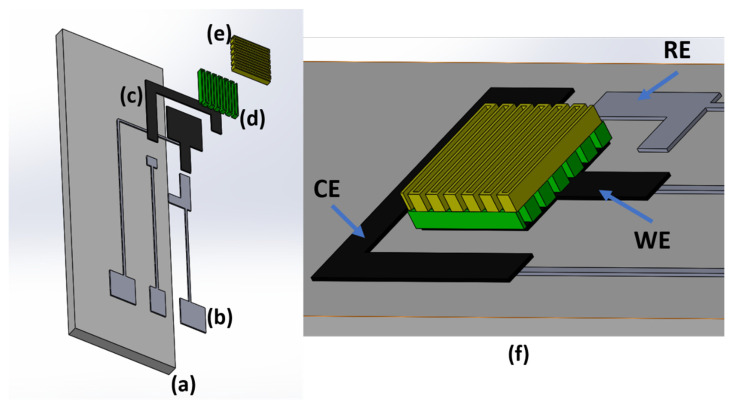
Render design of each layer of the sensor. Alumina substrate (**a**), AgCl conductive tracks (**b**), carbon coating (**c**), lines (**d**) and grid (**e**) microstructuration. (**f**) Stacked layers are depicted in detail. Unrealistic colors were chosen to enhance the visibility of each layer.

Considering this evidence from the literature, the proposed 3D electrodes were designed to provide a 15 mm^2^ WE base area and to fit in a 10 × 30 mm alumina substrate, compatible with a commercial connector for screen-printed electrodes. A two-material architecture was selected to provide good electrode conductibility, stable RE and inert CE and WE. According to those choices, silver chloride (AgCl ink, XA-3773, Fujikura Kasei Co. Ltd., Shibakouen Minato-ku, Tokyo, Japan) was employed for conductive tracks, pads, and RE, and carbon (C ink, EXP 2652-28, Creative Materials Inc., Ayer, MA, USA) ink for WE and CE coating and further microstructuration. A 3D rendering of the electrode structure is provided in Figure 1. 

**Figure 2:**
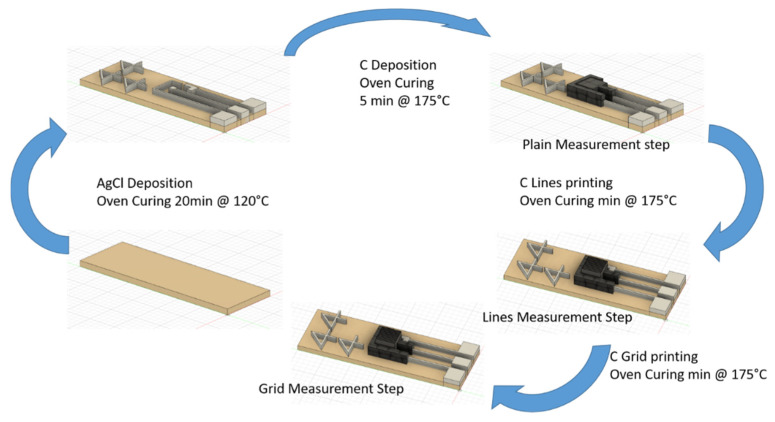
Electrode production process.

The sensors were produced using an aerosol jet printer (AJ300, Optomec, Albuquerque, NM, USA) in a five-step printing process shown in Figure 2. The substrates are initially cleaned in ethanol to remove possible contaminants and to increase the adhesion between the ink and substrate. Then AgCl ink is deposed and cured at 120 °C for 20 min. Next, WE and CE are coated with carbon ink and then cured at 175 °C for 5 min. These first steps were printed using a 750 μm head to coat the surface and obtain regular depositions, while the following fine functionalization lines were printed using a 200 μm head to achieve a line width around 100 μm. The functionalization was printed in two successive steps divided and followed by the ink curing. After the first microstructuration step, the second microstructuration layer was printed. 

**Figure 3:**
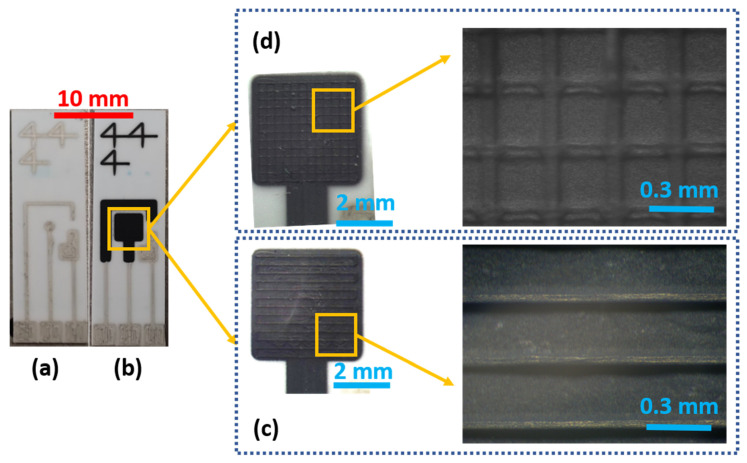
Step by step produced electrodes. (**a**) Silver Chloride conductive tracks and RE, (**b**) WE and CE carbon coating, (**c**) line microstructuration, (**d**) grid microstructuration.

To provide a correct alignment between the different layers, a set of three four-shaped fiducials was inserted (Figure 2). These were used to calculate the different positioning of the substrates during the production step and thus correct the printing path. Five sensors were produced after an accurate selection of the process parameters (reported in Table 1) such as substrate temperatures and process speeds that promote the inks deposition. In Figure 3, the output of each production step is depicted.

### 2.2. Physical and Electrochemical Evaluations

Electrical, morphological and electrochemical characteristics of the produced electrodes were evaluated after printing. The electrode resistances were measured using a 4-wire technique using a digital bench-top multimeter Hewlett–Packard 34401a (HP, Palo Alto, CA, USA) to evaluate the process variabilities. Geometrical features were evaluated using an Alpha-Step IQ Surface Profilometer (KLA-Tencor, Milpitas, CA, USA). Those first measurements aimed to evaluate the production process and identify drift and differences between electrodes. At first, we evaluated the profile of microstructured lines produced on bare alumina. After performing electrode microstructuration on 5 electrodes, their profiles were acquired, and statistical analysis was performed. Each of the five profiles is divided into 13 signals that depict each a single microstructured peak. Those signals were properly aligned and analyzed to obtain both the peak thickness and width. The former was evaluated as the mean of points that are at least 80% of the maximum acquired value. The average width was calculated as the distance between the points whose thickness was 10% of the maximum value. A similar evaluation was later performed on the produced electrodes on all two possible microstructurations. 

Electrochemical evaluations were performed using a portable potentiostat Palmsens3 EIS (Palmsens, Compact Electrochemical Interfaces, Houten, Utrecht, The Netherlands). All the electrochemical experiments were performed in a Phosphate Buffer Saline (PBS) solution (50 mM, pH 7.0) as a supporting electrolyte and using ferro/ferri-cyanide ([Fe(CN)_6_]^3−/4−^) as analyte. The latter is considered a classical redox probe due to its well-known redox parameters [26], low cost and ease of detection that makes it a standard analyte for electrode characterization. Different preliminary electrochemical experiments were performed on the materials to define the best potential window from zero to one volt [0; 1] V that allows the analyte detection without electrolyte interference. According to them, the measurement protocol was defined. Each session started applying a constant 1 V bias to WE in PBS for 120 s, thus electrochemically cleaning the surface by stripping any deposited salt. Then, 8 CVs (scan rate 0.1 V/s, voltage range [0; 1] V) were performed in PBS solution to stabilize the electrodes and acquire blank voltammograms. Those first two steps were performed as a pretreatment technique to enhance the electrochemical activity of the electrodes and remove contaminants [27]. In order to improve the sensitivity and reduce the effects of the charging current, differential pulse voltammetry (DPV, pulse amplitude 0.2 V, pulse time 0.01 s, scan rate 0.2 V/s) was used to evaluate five different solutions with increasing ferro/ferri-cyanide concentrations (0 mM, 2 mM, 4 mM, 6 mM and 8 mM). At the end of those measurements, a DPV and four CVs in PBS are performed to collect information on adsorption. The experiment was concluded with a stripping equal to the first one to clean the sensor. On each electrode, all measurements were performed three times, the first on sensors with bare carbon WE and CE, the second after printing parallel lines on top of bare electrodes and the last one after the complete grid deposition. This procedure allowed neglecting the inter-sensor variation and focused on the intra-sensor variation due to the different functionalization. All the acquired measures were later elaborated using MATLAB R2019b to perform statistical analysis, data fitting and to extract useful information on the overall behavior of the electrodes. 

## 3. Results and Discussion

### 3.1. Physical Evaluations

**Figure 4:**
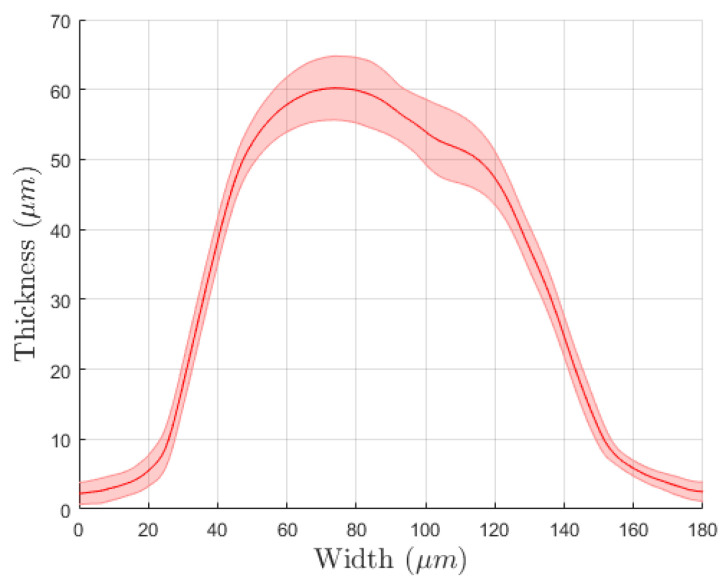
Microstructuration profile obtained for test microstructuration on bare alumina. The solid line depicts the average thickness of the peak while the shaded area represents the standard deviation. That statistical information was obtained from 60 sample peaks.

**Figure 5:**
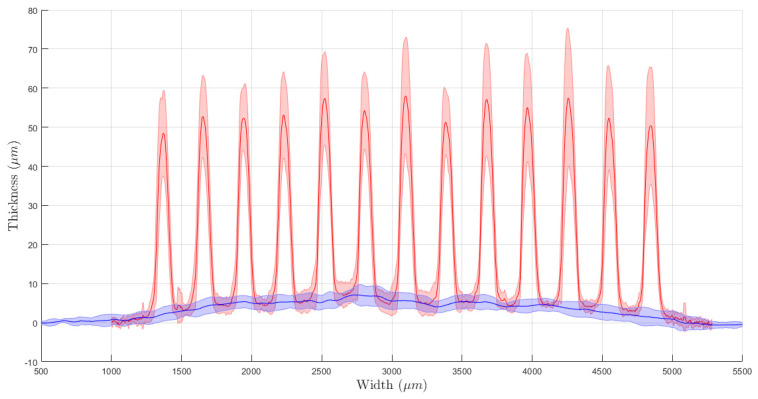
Average profiles of the WE of the produced electrodes. In blue, the bare WE are presented, while the microstructures are represented in red. As before, solid lines represent the average thickness, and shaded areas represent the standard deviation.

**Figure 6:**
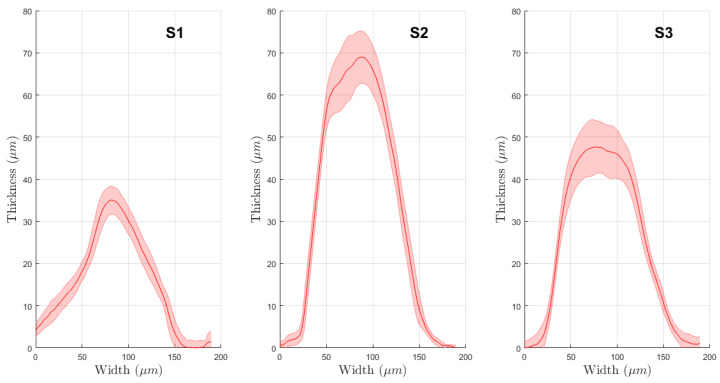
Average profiles obtained on different electrodes after the microstructuration.

The physical evaluations allowed characterizing the production process and evaluate their effects on the electrochemical performances. During the first two deposition steps, the resistance of each electrode was measured. The silver–silver chloride tracks had different values according to their length. The average resistances were (15.44 ± 4.11) Ω for CE, (8.63 ± 2.50) Ω for WE and (6.97 ± 2.35) Ω for RE. The set of profile evaluations acquired after each deposition showed a low variability in the production process. In detail, non-microstructured WE resulted in (5.27 ± 0.76) µm thickness on average, with a mean width of (4.59 ± 0.70) mm. This ensures good coverage of the silver layer and guarantees a correct behavior of the electrodes preventing metal oxidation. In Figure 4, the average profile for the test line printed on the bare alumina is depicted. They present an average thickness of (58.20 ± 1.85) µm and an average width of (132.78 ± 5.67) µm. After this evaluation step, we printed the microstructure directly on the WE surface, whose profile is shown in Figure 5. Analyzing the profile of those peaks, we observed a similar average peak width of (130.09 ± 21.22) µm and a smaller thickness of (48.87 ± 13.86) µm. The comparison between subsequent deposition processes showed an increased relative standard deviation that suggests variability and drift in the deposition process due to the serial printing (the electrodes were printed one after another). Those differences can also be observed by analyzing the mean profile shape obtained on a single electrode. In Figure 6, it is possible to observe different peak profiles that are due to fluctuations in the process parameters. Similar phenomena were also observed on the grid microstructures. In this last situation, we obtained a (19.57 ± 8.71) µm average peak thickness and a (131.02 ± 19.37) µm average peak width. 

### 3.2. Preliminary Electrochemical Tests

**Figure 7:**
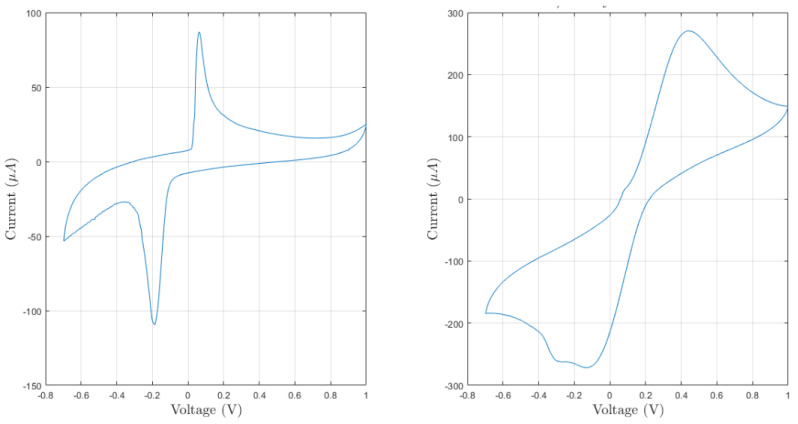
Voltammograms obtained after two experiments performed in pure PBS (**left**) and in 8 mM ferro/ferricyanide solution (**right**) with a scan rate of 200 mV s^−1^.

**Figure 8:**
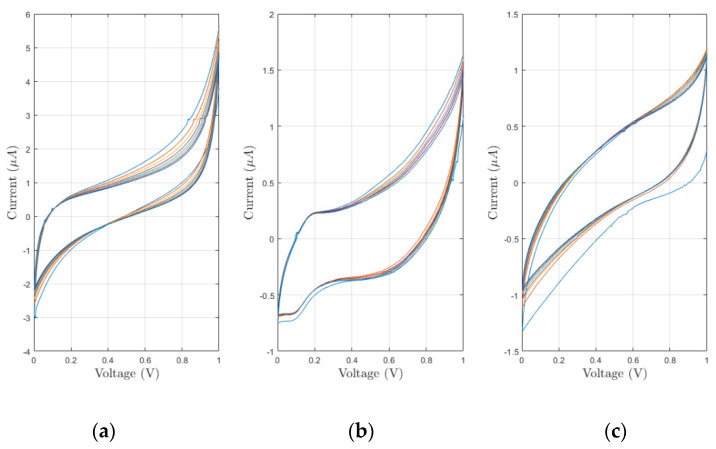
Voltammograms obtained by experiments performed in pure PBS for bare (**a**), line microstructured (**b**) and grid microstructured (**c**) sensors.

Preliminary experiments were performed to evaluate the feasibility of our approach and to determine important electrode’s characteristics. At first, different cyclic voltammetry (CV) experiments were performed both in pure PBS as blank solution and in ferro/ferricyanide solution. As reported in Figure 7, CV produced by the ferro/ferricyanide solution present two well-distinct peaks at 0.45V and −0.1 V. As regards the CV produced by the blank solution, they present two spurious peaks at around 0.1 V and −0.2 V. Different potential windows were tested to minimize the impact of the undesired peaks. In the end, a [0; 1] V potential window was chosen because it suppresses all the unwanted peaks and allows identifying the oxidative peak of the analyte of interest. In Figure 8, eight CVs obtained in blank solution for each sensor’s microstructuration are reported. CVs present similar peak currents and shapes. Moreover, a quick stabilization after only few cycles can be appreciated. 

### 3.3. Electrochemical Evaluations

All five sensors were tested using the aforementioned protocol that starts comparing the reaction of the three possible microstructurations in a PBS blank solution. All the electrodes with all the microstructurations produce with DPVs similar blank electrochemical behavior, with a plain region in the [0.1; 0.7] V potential window. This is convenient in our application, where we expect from previous assessments, a peak due to the ferro/ferricyanide reaction around 0.5 V. 

**Figure 9:**
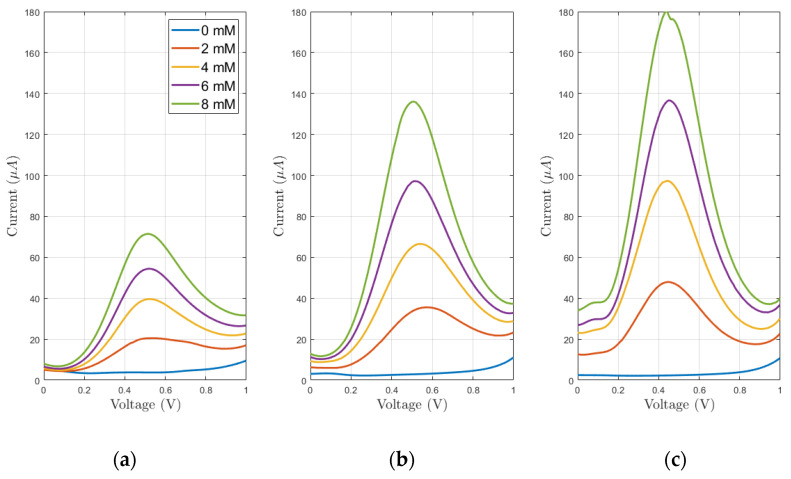
DPVs obtained on sensor S2 for the bare electrodes (**a**), lines (**b**) and grid (**c**) microstructured ones.

**Figure 10:**
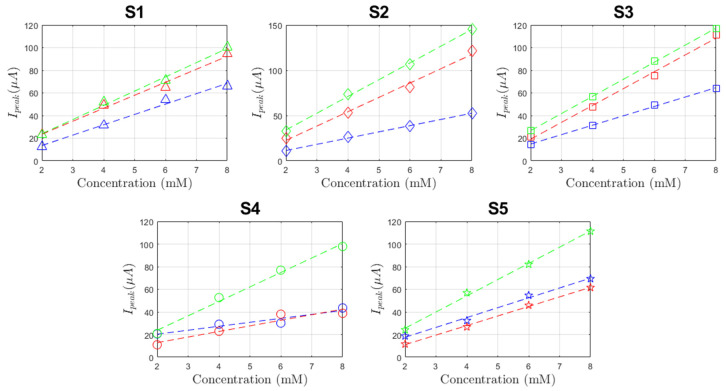
Sensor calibration points for bare (blue), line-microstructured (red) and grid-microstructured (green) electrodes. Dashed lines represent the best fit lines for each configuration.

**Figure 11:**
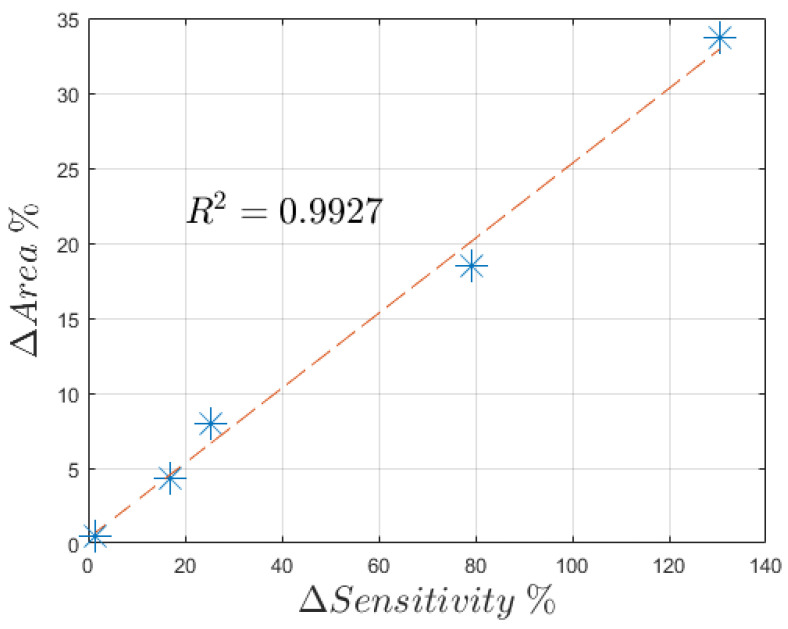
The obtained relationship between the microstructuration-added electrode area and the increase in sensitivity. The blue asterisks represent the data points, and the dashed red line is the best fit line that presents *R*^2^ = 0.9927. The data for the added area were extracted from the profiles presented in Figure 6.

In Figure 9, the data collected from DPV performed on each configuration (bare, lines and grid microstructuration) on a single sensor are reported. There, it is possible to observe an overall improvement of the peak current for each concentration. This implies ease of detection of the analyte using microstructured devices rather than through non-microstructured ones. To further inquire about the sensitivity of our devices, the peak width was extracted through the PSTrace 5.8 software that provides both the peak current and position. The latter, expressed as peak voltage, is stable for all three configurations, resulting as slightly higher on average for plane electrodes at (504.65 ± 16.75) mV, rather than on line-microstructured (479.75 ± 37.94) mV and grid-microstructured (468.94 ± 22.92) mV electrodes. As regards the peak currents, each sensor was compared separately. In Figure 10, the peak currents are related to the analyte concentration to obtain three calibration curves for each sensor. Those curves resulted as linear as expected from the theoretical background [24,25]. Considering the sensitivity as the slope of the fitting line, it was shown that grid microstructures deposition improved the sensitivity with respect to the bare devices on average of 2.3 times, while the line microstructures produced an improvement of 1.5 on average. It was also shown that those microstructurations do not interfere with the linearity of the device (the linear fit produced an *R*^2^ > 0.98). The variability among the different sensors is due to the multiple variables that influence the electrochemical process. For example, the differences of the obtained microstructured profiles play a major role in defining the different changes in the sensitivity of the sensors. In detail, it resulted that the line microstructuration for sensor S1 produced a 25% improvement of the sensitivity with an average peak thickness around 35 µm and an estimated added area of 8%, while sensor S2 experienced a 130% variation due to peaks up to 70 µm that produced an estimated added area of 33%. These data present (Figure 11) a linear dependence between the percentual increase of the slope and the one of area, as already foreseen by Equations (1) and (2).

## 4. Conclusions

In this work, we presented a promising methodology to improve the sensitivity of electrochemical sensors by exploring a third-dimension microstructuration in fully printed devices realized by aerosol jet printing. Here, we were able to obtain a linear structure up to 70 μm thick with an average line width around 130 μm. The microstructures permit increasing the surface up to 130% without changing the substrate occupancy. Our tests revealed that the microstructuration process was able to increase the sensitivity of the electrodes on average of 2.3 times with respect to the bare electrodes. 

The proposed microstructuration can be adopted in applications that require high sensitivity. Moreover, those geometrical features are comparable with the ones obtained in other works [14,17,18] with lithographic processes instead of the hereby proposed fully additive, digital and maskless production process, thus allowing greater flexibility and opening to new possible geometries. 

Future work will mainly address the improvement of the high process variability obtained in this preliminary work by optimizing each production step, for instance, parallelizing the deposition and reducing the production time. Moreover, further work will experimentally inquire the limits of this approach in terms of maximum added area, considering both the microstructure spacing and their thickness. In particular, the latter is expected to present limitations due to the repeated deposition process that could modify the thickness–width ration and the shape of the microstructures, limiting thus the effective improvement of sensitivity.

## Figures and Tables

**Table 1:** Printing parameters used during the production process.

	Ink	Sheath Flow (SCCM)	Atm Flow (SCCM)	Exhaust Flow (SCCM)	Substrate Temperature	PROCESS SPEED	Number of Depositions
Conductive tracks, RE	AgCl	250	1100	1030	50 °C	3 mm/s	1
CE and WE coating	C	400	1150	1030	75 °C	3 mm/s	6
Microstructuration	C	40	805	790	70 °C	2 mm/s	20

## Data Availability

Data are available upon request to the authors.
